# Co‐Designing a Care Pathway for Healthcare Professionals to Recognise and Respond to Human Trafficking Victims in Emergency Departments

**DOI:** 10.1111/hex.70709

**Published:** 2026-05-29

**Authors:** Leanne van Rooy, Celia J. Filmalter

**Affiliations:** ^1^ Department of Nursing Science School of Health Care Sciences University of Pretoria Pretoria South Africa

**Keywords:** care pathway, emergency department, experience‐based co‐design, healthcare professionals, human trafficking survivors

## Abstract

**Background:**

Victims of human trafficking frequently present to emergency departments (EDs) while being exploited; however, many remain unrecognised by healthcare professionals. Creating spaces where survivors and healthcare professionals can engage provided a unique opportunity to generate a care pathway which increases the likelihood of implementation as the needs and expectations of the victim and healthcare professionals are considered.

**Objective:**

To describe the process and outcome of experience‐based co‐design of a care pathway that enables healthcare professionals to recognise and respond to human trafficking victims in EDs.

**Design:**

Experience‐based co‐design.

**Setting and Participants:**

Twenty‐seven individuals participated in two co‐design events. The in‐person event included six registered nurses, one social worker, three trauma counsellors, one psychologist and four survivors. Twelve participants took part in a virtual event, comprising six medical doctors, two pre‐hospital medical personnel, one counsellor, one psychologist and two survivors.

**Intervention:**

Data from the co‐design events were analysed using a creative hermeneutic approach. Co‐designers generated the care pathway using data from a mapping review, interviews with victims of human trafficking and focus groups with healthcare professionals.

**Results:**

The in‐person group identified five themes, including education, safety, alertness, compassion, nurturing and action. The online event highlighted information sharing, special victim champion, safety and security, primary response and awareness. The themes were combined into three main themes, namely education and awareness, safety and security and human disposition, underpinned by compassion, non‐judgement, trustworthiness and trauma‐informed care.

**Conclusions:**

The co‐designed care pathway prioritises education and awareness to help healthcare professionals recognise and respond to potential trafficking victims in EDs. The care process can be initiated using ‘Code Orange’, supporting coordinated, interprofessional and comprehensive interventions. Using a person‐centred, trauma‐informed approach, the care pathway emphasises safety, security and compassion for both victims and healthcare professionals.

**Patient or Public Contribution:**

Survivors and healthcare professionals co‐designed the care pathway.

## 1 Introduction

Human trafficking is a public health problem [1]. Survivors have reported that 60%–80% of human trafficking victims visit emergency departments (EDs) while being exploited, creating opportunities for recognition [2, 3, 4]. However, approximately 87% of victims remain unrecognised during ED visits [2, 3, 5]. In EDs, healthcare professionals play a vital role in detecting victims, and a care pathway is needed to provide basic medical and psychological support [3].

Care pathways are complex interventions that support collaborative decision‐making and organised management for a specific group of patients in specific situations [6]. Care pathways promote standardised clinical practice by outlining the optimal sequence and timing of interventions for particular patient groups [7]. Care pathways are evidence‐based tools designed to guide clinical decision‐making, especially for patient groups that require targeted interventions, such as victims of human trafficking [8, 9].

Standardised care pathways enhance professionalism, especially when incorporated into supportive organisational environments [8]. Care pathways also promote ongoing education and training for healthcare professionals, thereby contributing to continuous professional development [10]. This ensures that healthcare professionals remain current with evidence‐informed practices, improve their clinical knowledge and practical skills and increase their ability to recognise and respond to targeted populations [10, 11]. By integrating training into the care pathway, healthcare professionals are supported in maintaining their competence and fostering better interprofessional collaboration, effective communication and overall patient outcomes [7, 10, 11].

Additionally, care pathways promote coherence and consistency within interprofessional teams, empowering patients through shared decision‐making [9]. This improves communication between healthcare professionals and patients, ensuring coordinated care, shared accountability within the interprofessional team and role clarification [7, 10, 11]. Given the complex and often hidden nature of human trafficking, a standardised care pathway co‐designed by patients (victims) and healthcare professionals is required [12, 13, 14].

Co‐design is the collaboration among people who actively participate and work toward a common goal, such as developing a care pathway [15, 16, 17]. The co‐design process encourages the exchange of diverse perspectives and knowledge, allowing for constructive and creative dialogue [17, 18]. The collaborative process supports co‐creation and promotes systemic change through partnerships with survivors and healthcare professionals [16, 17].

This article describes the process and outcome of experience‐based co‐design (EBCD) of a care pathway that aims to enable the recognition and response to human trafficking victims in EDs by healthcare professionals. The study was conducted in the EDs of one private hospital group in Gauteng, South Africa.

## 2 Methods

### 2.1 Design

**Figure 1:**
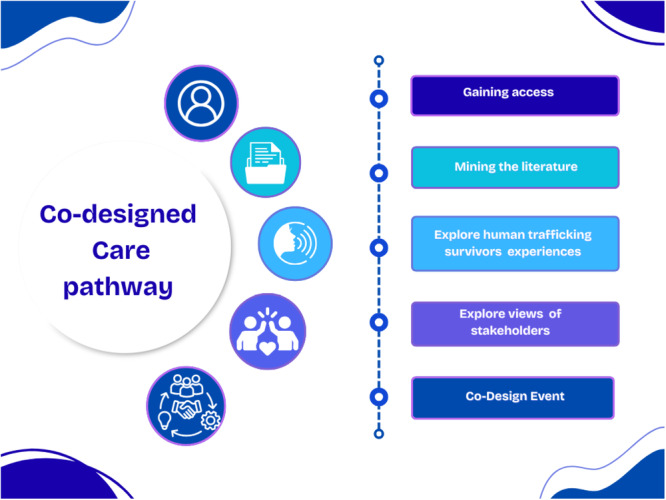
Steps of the experience‐based co‐design (EBCD) process.

The EBCD event formed the final part of a larger study that applied a multi‐method research design using three stages, namely, a mapping review to identify key elements recommended for inclusion in a care pathway. This was followed by exploring the experiences of survivors through semi‐structured individual interviews (*N* = 12) and focus groups with healthcare professionals (*N* = 3) using both in‐person and online methods. The EBCD methodology of Bate and Robert [19] was employed to enhance the quality, responsiveness and human‐centredness of care desired by victims of human trafficking admitted to EDs. Ensuring these improvements are rooted in participants' authentic experiences, stakeholder insights and a culture of continuous learning and collaborative innovation [16, 20]. The audio recordings from the survivors' semi‐structured interviews were collated by a professional specialising in video production, who created a 10‐min AI‐generated video using computer‐generated avatars and voice blurring, highlighting key touchpoints identified by survivors (see Appendix 1 for QR code to 10‐min video). The video producers signed confidentiality clauses. The authors remained committed to beneficence, confidentiality and respect for individuals throughout the study. The purpose of the video was to capture the emotional essence of the participants' situations and represented the central element of EBCD [16, 20]. The video served as the catalyst in the co‐design event, allowing participants to visualise survivors' stories and creating an emotional and cognitive foundation for discussions [21]. Figure 1 depicts the steps involved in the co‐design process.

### 2.2 Setting

The in‐person co‐design event was held in a lecture room at a selected hospital. A second co‐design event was conducted online using Microsoft Teams to accommodate participants who could not attend the in‐person event due to location. The researcher negotiated and scheduled dates and times that suited the participants. The participants included those involved in preceding data collection sessions that indicated their willingness to contribute in the EBCD event.

### 2.3 Participants

Twenty‐seven individuals participated in the co‐design event. All healthcare professionals had more than 5 years of experience in ED, and some had worked with victims. During the in‐person event, 15 individuals attended, including six registered nurses, one social worker, three trauma counsellors, one psychologist and four survivors.

Participants who indicated their willingness to participate in the EBCD event were invited via WhatsApp. The venue was selected at a centrally located hospital for both survivors and stakeholders. Three tables with stationery were set up, and refreshments were provided during the 3‐hour event. The researcher offered the survivors the option to arrive early and watch a 10‐min video of survivor stories before the event, and all four survivors agreed. The two survivors attending the online event were invited to view the 10‐min video in advance; however, both preferred watching it during the session rather than beforehand. This did not negatively impact their participation; both actively engaged in the session, contributed meaningfully and were fully involved in the discussions.

The online event lasted for 2 hours, with participants joining the session from their homes or offices in private rooms. Twelve participants participated in the virtual co‐design session, including six medical doctors, two emergency services personnel, one counsellor, one psychologist and two survivors.

### 2.4 Survivor Involvement

Survivors shared their stories of being trafficked and receiving care in EDs. Their lived experiences and reflections added authenticity and depth to the development of the care pathway. Importantly, all survivors actively participated in initiatives aimed at helping and empowering other victims and survivors of human trafficking. Healthcare professionals also participated as co‐designers of the care pathway. The discussions examined key components of the care pathway and, importantly, how victims preferred to be treated in EDs.

### 2.5 Co‐Design Event

The co‐design event used creative hermeneutic data analysis, which allows participants to move beyond descriptive accounts to identify underlying meanings and key emotional touchpoints within their narratives, supporting a nuanced, experience‐driven foundation for the co‐design care pathway [22].

**Figure 2:**
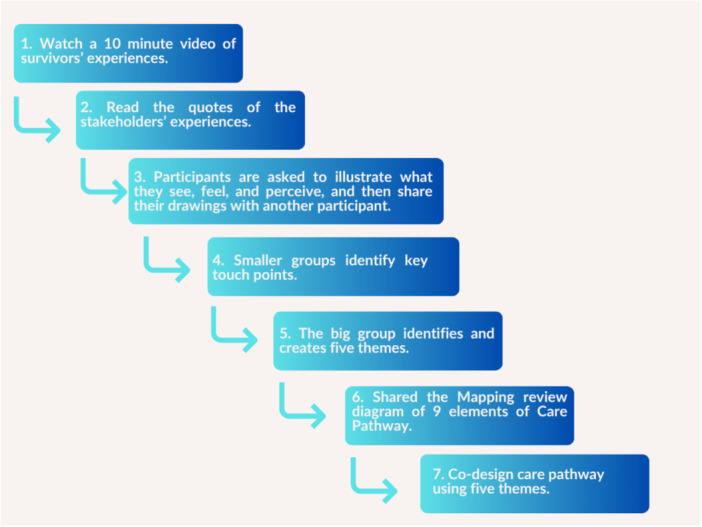
Creative hermeneutic data analysis steps used during the co‐design event [21].

The creative hermeneutic data analysis method and steps suggested by were followed to create a preliminary care pathway (Figure 2).

After obtaining informed consent, the AI‐generated video was shown (see Appendix 1 for QR code to 10 min video), followed by sharing the data from the preceding data collection sessions with the participants and allowed 15 min to read and review the findings. Each data set was divided into two sections with two participants reviewing the same set of data to enhance the reliability of the touchpoints.

The participants were then divided into smaller groups, where each participant created images of shared stories and experiences. The small groups identified key touchpoints based on stories and discussions. Key touchpoints are defined as interactions between survivors and healthcare professionals that are perceived as important for recognising and responding to victims in EDs [25]. Each group wrote agreed‐upon touchpoints on strips of paper. The groups were then combined into one, sharing the written touchpoints, which were placed on the floor in the middle of the group (for in‐person events) and on the Excalidraw Whiteboard (https://excalidraw.com/) for the online event. After discussions and clarifications, the entire group reached consensus on the key touchpoints that represent the core elements of the pathway. Five preliminary themes were identified from each group, and the group co‐designed a draft care pathway. The same process was followed for online events.

### 2.6 Ethical Considerations

The study was approved by the xxx (711/2023), and all participants provided written informed consent. Owing to the sensitive nature of the data and the vulnerability of the participants, the Research Ethics Committee did not allow audio recordings during the co‐design events involving survivors. Survivors shared personal details that could jeopardise their safety. A trauma‐informed approach was part of the bigger study, which included creating a safe environment, building trust, being transparent, empowering survivors to make their own choices and respecting diversity [23]. No remuneration was provided to survivors; however, transport costs for the in‐person event and data expenses for online participation were covered. At all times, a counsellor and psychologist were available to assist the survivor's needs for psychological support.

**Figure A1:**
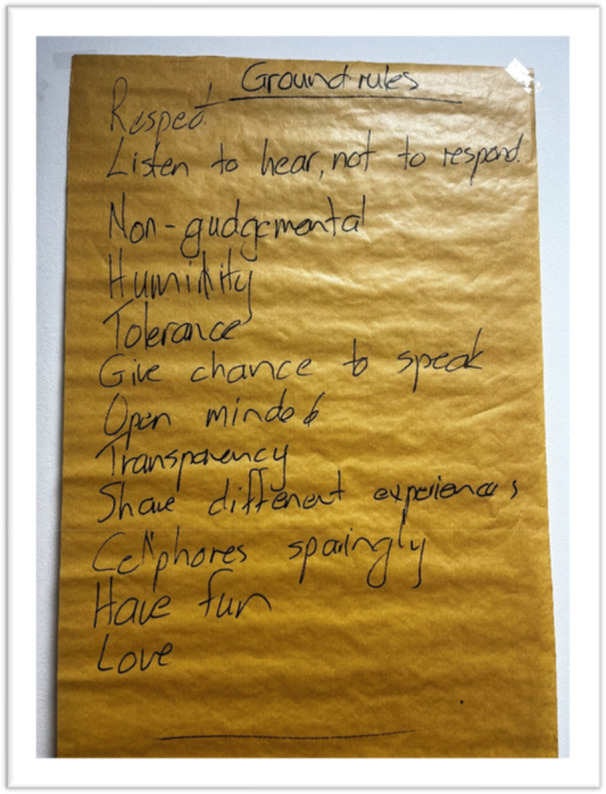
Ground rules for in‐person event.

External validation was supported by engaging an independent facilitator who facilitated the co‐design process, helping minimise researcher influence [24]. Member checking further enhanced credibility, as both survivors and healthcare professionals actively reviewed, validated and refined key touchpoints [24]. Rules of engagement were established prior to the session, ensuring that all participants had an equal opportunity to contribute. Power dynamics were actively managed (Appendix 1, Figure A1). Ground rules included mutual respect, listening to understand rather than to respond, non‐judgement, humanity, tolerance, allowing others the opportunity to speak, sharing diverse experiences and fostering an open, engaging environment.

## 3 Results

The in‐person group yielded five themes, namely, education, safety, alertness, compassion, nurturing and action (Table 1). The online event featured themes including information sharing, special victim champion, safety and security, primary response and awareness (Table 1). The authors and co‐design facilitator engaged in reflective discussions to gain consensus on the main themes. These themes were merged into three themes: education and awareness, safety and security and human disposition, underpinned by compassion, non‐judgement, trustworthiness and trauma‐informed care (Table 2).

**Table 1:** Themes and touchpoints identified at the in‐person and online event.

In‐person event		Online event	
Theme	Touchpoint	Theme	Touchpoint
Education	Education and training	Awareness	Education and awareness
	Professional etiquette		Hotline information
	Frequent flyers		Trauma‐informed care
Safety	Security	Safety and security	Trustworthy staff
	The Protection of Personal Information Act (POPIa)		Staff safety
Alert	Screening tools		General workplace culture and reporting channels
	Probing questions	Primary response	Exit strategy
	SOS code		Private consulting rooms
	Triage		Policies
Compassion and nurturing	Safe space		Victim identification
	Sensitivity with culture, language and gender		Alert
	Recognition		Screening tools
	Non‐judgemental		Continuous suspicion
Action	Chain of survival	Information sharing	Bathroom poster
	Holistic approach		‘See something, do something’
	Policies and procedures	Special victim champion	

**Table 2:** Themes and key touchpoints for care pathway.

Theme	Key touchpoint
Education and awareness	Triage
	Self‐identification
	Screening tools
	Posters with hotline number
Safety and security	Private and secure rooms
	Safety response plan
	Reporting channels
Human disposition	Compassion
	Non‐judgement
	Trustworthiness
	Trauma‐informed care

Participants in both events emphasised the importance of a care pathway, focusing on key touchpoints, such as education and training for healthcare professionals on human trafficking and on raising awareness. Healthcare professionals need to know about the ‘red flags’ related to human trafficking, legislation regarding their roles and responsibilities and referral procedures. Participants stressed the importance of recognising specific clinical patterns that could indicate potential trafficking. The use of screening tools during triage, the activation of an alert system named ‘Code Orange’, and corrective actions were discussed. One participant proposed fostering a ‘see something and do something’ culture among healthcare professionals to prompt alerts if they suspect a victim. Another suggestion was to place posters in EDs about human trafficking, including the hotline number, especially in private areas, such as restrooms, and to provide instructions for alerting healthcare professionals, by marking specimen bottles with a specific colour pen to promote self‐identification. Additionally, participants proposed appointing a ‘special victims champion’ responsible for activating and coordinating the ‘Code Orange’, which entails notifying the interdisciplinary team, including security, police, social workers and contacting the Human Trafficking Hotline.

A key touchpoint was the human disposition of healthcare professionals, who should respond with compassion and a non‐judgemental, trauma‐informed approach. Survivors mentioned that traffickers may bribe and intimidate healthcare professionals to prevent reporting. The trustworthiness of healthcare professionals is thus a key touchpoint. The participants agreed that a safe workplace culture and clear reporting channels are essential, with hospital security being involved when necessary. All participants agreed that the interprofessional team should follow a holistic approach to ensure a chain of survival with appropriate healing support for survivors.

The safety of both human trafficking victims and healthcare professionals was a key touchpoint. The participants highlighted the importance of private consulting rooms. Participants noted that healthcare professionals often feel unsettled by human trafficking victims because of limited knowledge and resources. Additionally, healthcare professionals often feel troubled as traffickers may become violent. Participants suggested that victims should have an exit strategy. An essential safety measure includes protecting the personal information of trafficking victims. The hospital security team was identified as a vital part of managing the victims of human trafficking. The participants discussed the necessary security protocols and the importance of creating a safe emotional space for victims to disclose their status.

## 4 Discussion

**Figure 3:**
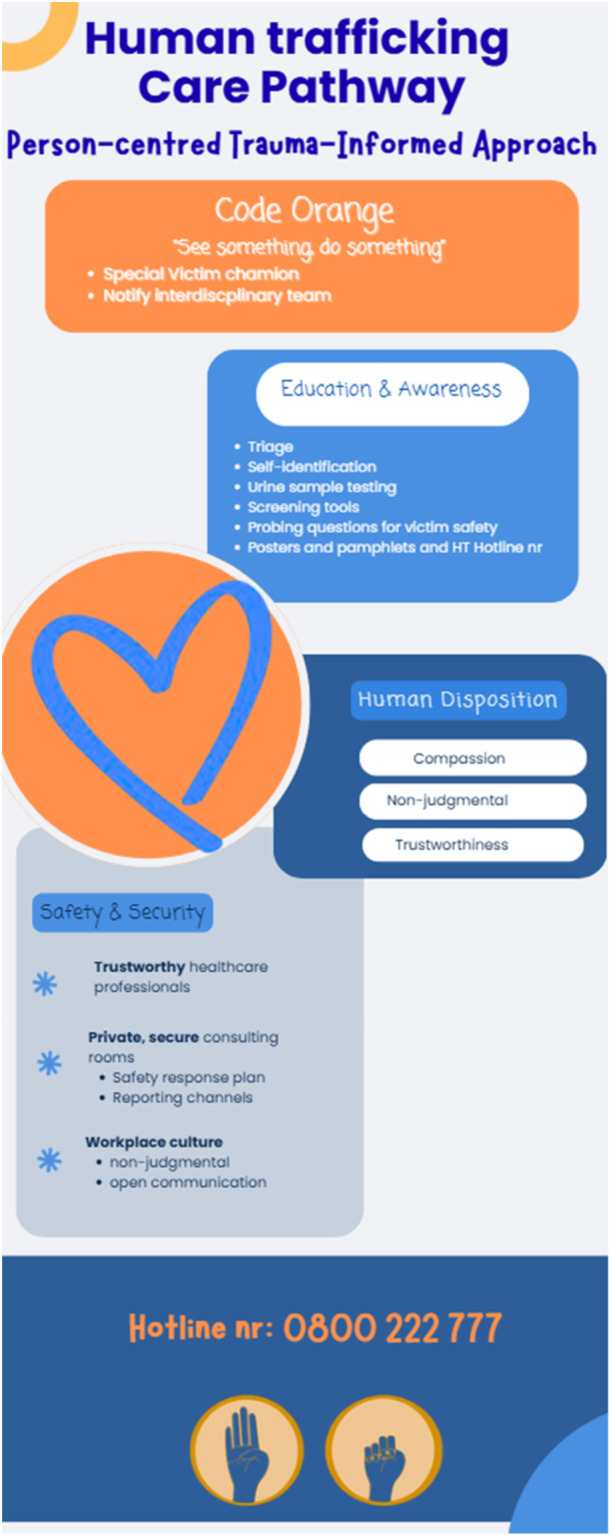
Care pathway for healthcare professionals to recognise and respond to human trafficking victims in emergency departments.

This study used an EBCD methodology to develop a care pathway for victims of human trafficking admitted to EDs (Figure 3). The ‘Code Orange’ is an alert system designed to promote a culture of ‘see something, do something’. The care pathway positions healthcare professionals as key decision‐makers who integrate clinical expertise, contextual needs and survivor‐centred priorities [26, 27]. Education and awareness are fundamental to guide healthcare professionals in identifying and coordinate interdisciplinary team response to potential trafficking victims. The identification of victims starts with triage and initial contact. Triage nurses play a key role in the initial identification process [28, 29, 30, 31]. Healthcare professionals should therefore be trained to identify red flags to enable early detection and improve care [2, 4, 28, 29, 32, 33].

Robust actions are necessary to facilitate opportunities for the identification of potential victims. Healthcare professionals can use probing questions to assess a victim's safety, allowing sufficient time for discussion and facilitating connections to appropriate support resources, in line with the principles outlined in the HEAL Toolkit [14, 33]. Participants suggested that urine sample testing may present such an opportunity. Victims can self‐identify by placing an orange dot on a specimen bottle in the bathroom, and then the triage nurse will take the victim to a private room. This practice is common in EDs across the United States [2]. Posters and pamphlets can also be used to provide information on resources and hotline numbers, supporting subtle actions in EDs.

Assigning a ‘special victim champion’ responsible for activating and coordinating ‘Code Orange’ may enable comprehensive care through an interprofessional team approach, while facilitating safety and security measures to protect both victims and healthcare professionals. Through the ‘special victim champion’, safe spaces that is sensitive to gender, culture and language can be arranged. Many victims are afraid of self‐identifying due to fear of traffickers and being judged, highlighting the importance of respecting diversity and equity [2, 5]. The trust relationship between victims and healthcare professionals is essential for victims to disclose their status. This was clearly described by survivors who participated in the EBCD process. Victims are often unable to disclose their status because they are controlled by traffickers [26, 33]. Participants, especially survivors, emphasised the need for private consulting rooms. The ED environment should offer private, secure consultation rooms for disclosure, with a safety response plan in place. Participants identified the need for an exit plan and reporting channels supported by the PEARR tool [33].

The importance of human disposition was highlighted during the EBCD process stressing that a person‐centred trauma‐informed approach should always be used. Healthcare professionals should engage with victims in a non‐judgemental and compassionate way while actively listening to victims [31, 34]. A professional and supportive culture is needed to mitigate risks, protect and promote the quality of care provided to victims [35]. Victims of human trafficking experience unique trauma, which adds to secondary trauma that healthcare professionals might experience [35]. Healthcare professionals should be supported to ensure their mental well‐being. The safety of healthcare professionals and survivors remains a priority. Healthcare professionals who are dealing with human trafficking victims often fear traffickers and may choose to ignore the problem [3].

### 4.1 Strengths and Limitations

The in‐person event was limited by time constraints, which reduced the depth of engagement, particularly for survivors, whose contributions were central to the co‐design approach. Several limitations of the online event included participants disengaging before the session ended; some were hesitant to contribute at first, and technical challenges, such as poor audio quality and platform issues, disrupted communication and reduced the efficiency of facilitation. The drawing activity exceeded the allocated time, echoing the known time‐intensive nature of EBCD processes and illustrating how easily engagement can decline when activities run longer than expected. These issues reinforce the concern that EBCD may be difficult to implement in resource‐constrained or high‐pressure environments [36].

Nonetheless, both events highlighted several strengths of EBCD. Survivors and healthcare professionals appreciated the opportunity to deepen their mutual understanding, recognise each other's perspectives and co‐develop a care pathway for EDs. These results demonstrate the relational and experiential benefits of EBCD. Participants described the environment as respectful, inclusive and collaborative, supporting the elevation of survivor voices and aligning closely with the EBCD's focus on shared decision‐making.

Importantly, this study, to the researchers' knowledge, represents the first attempt to conduct an online co‐design event in this context. This further highlights the need to adapt EBCD methods to various settings, while acknowledging that such adaptations may introduce additional limitations not present in traditional in‐person EBCD models. To enhance co‐design in an online setting, facilitators and participants should become familiar with the chosen digital tools (e.g., Excalidraw) prior to the session. Plan for about 4 hours for the session and increase the use of breakout rooms on platforms like Microsoft Teams to promote smaller‐group interactions. Adding interactive elements, such as allowing participants to actively contribute and draw on a shared online board, can further boost engagement and collaboration.

### 4.2 Recommendations

The authors recommend implementing the care pathway in EDs with targeted training for healthcare professionals and the adoption of a standardised, discreet screening method. This involves placing an orange dot on a urine sample, acting as a silent signal of possible human trafficking concern, prompting activation of a ‘Code Orange’ response. This method helps ensure safe identification while reducing risk to the patient.

The effectiveness of the care pathway can be evaluated by adherence to the screening protocol and the frequency of ‘Code Orange’ activations. Including the number of suspected victims identified, the appropriateness and timeliness of referrals to multidisciplinary support services and healthcare professionals' confidence in managing potential victim cases, along with feedback from healthcare professionals, will enable ongoing refinement of the pathway.

## 5 Conclusion

This collaborative care pathway prioritises education and awareness to help healthcare professionals in the ED recognise and respond to potential victims of trafficking. ‘Code Orange’ is a structured alert to initiate the care process, supporting coordinated, interprofessional and comprehensive interventions. Based on a person‐centred and trauma‐informed approach, the pathway emphasises safety, security and compassion for both victims and healthcare professionals.

## Author Contributions

**Leanne van Rooy:** conceptualization, writing – original draft, methodology, visualization. **Celia J. Filmalter:** conceptualization, methodology, writing – review and editing, supervision.

## Funding

The authors have nothing to report.

## Ethics Statement

Ethical approval has been obtained from the Research and Ethics Committee of the University of Pretoria (711/2023).

## Conflicts of Interest

The authors declare no conflicts of interest.

## Figures and Tables

**Figure A2:**
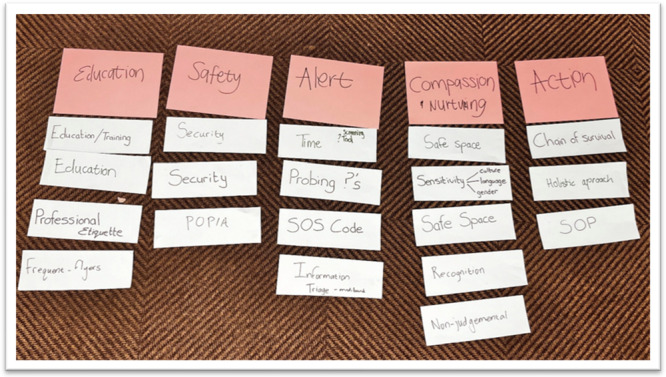
Five themes were identified at the in‐person event.

**Figure A3:**
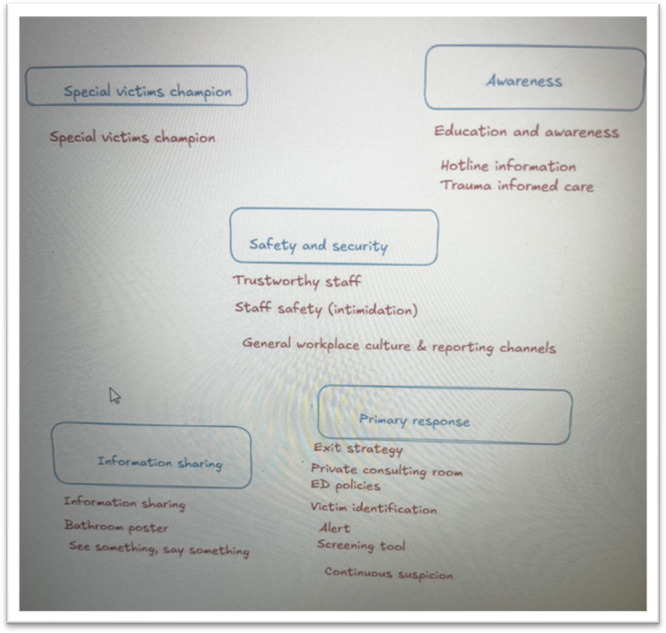
Five themes were identified at the online event.

## Data Availability

The data that support the findings of this study are available on request from the corresponding author. The data are not publicly available due to privacy or ethical restrictions.

## Appendix 1 Link to 10‐Minute Survivor Stories. Scan the QR Code

Figure A1, Figure A2, Figure A3
